# Checklist and distribution maps of the blow flies of Venezuela (Diptera, Calliphoridae, Mesembrinellidae)

**DOI:** 10.3897/zookeys.645.6972

**Published:** 2017-01-13

**Authors:** Yelitza Velásquez, Ana Isabel Martínez-Sánchez, Arianna Thomas, Santos Rojo

**Affiliations:** 1 Department of Environmental Sciences and Natural Resources, University of Alicante, E-03080 Alicante, Spain; 2 Laboratory of Biology of Organisms, Center for Ecology, Venezuelan Institute of Scientific Research, P.O. Box 20632, Caracas 1020-A, Venezuela

**Keywords:** Calliphorids, diversity, Neotropical Region, South America

## Abstract

A checklist of the 39 species of blow flies (Calliphoridae and Mesembrinellidae) so far known to occur in Venezuela is provided, based on a thorough literature review and the examination of ca. 500 specimens deposited in the main entomological collections of the country. Data from the literature and museum collections were used to generate distribution maps for 37 species. Three species are recorded from Venezuela for the first time: *Chrysomya putoria* (Wiedemann, 1830), *Mesembrinella spicata* Aldrich, 1925 and *Mesembrinella umbrosa* Aldrich, 1922.

## Introduction

Blow flies (also known as bluebottles, greenbottles, cluster flies and generically referred to as carrion flies) is the vernacular name traditionally used for the para/polyphyletic family Calliphoridae *sensu lato*. Historically, the taxonomic composition and phylogenetic relationships within this group of flies, belonging to the superfamily Oestroidea, have been controversial. During the last two decades, a division of Calliphoridae into 14 subfamilies has been widely accepted (Rognes 1997, Norris 1999, Kutty et al. 2010), even though some of these subfamilies are considered by many authors as independent families. This is the case of Mesembrinellidae (Kutty et al. 2010, Singh and Wells 2013, Marinho et al. 2016) and Rhiniidae (Kutty et al. 2010, Pape et al. 2011, Marinho et al. 2016), two taxa now widely ranked at the family level. However, not all studies support the same family/subfamily ranks, and Polleninae have been recently proposed as a family based on their phylogenetic position as sister group of Tachinidae (Singh and Wells 2013). Another group, Bengaliinae, has also been suggested as an independent family (Lehrer 2003), but further studies are required to support this controversial proposal and it currently remains widely accepted as a subfamily closely related to Auchmeromyiinae (Rognes 2005, Marinho et al. 2012). In this paper, we use the common name of blow flies to designate the traditional, non-monophyletic concept of Calliphoridae *s. l.*, whereas the term Calliphoridae is used to refer to a less inclusive taxon not containing Mesembrinellidae and Rhiniidae, which are nowadays generally accepted as separate families.

A single species of Rhiniidae, *Stomorhina lunata* (Fabricius, 1805), is present in the New World where it is found only on the island of Bermuda (Rognes 1991). Mesembrinellidae are a relatively small family of Neotropical blow flies occurring from southern Mexico to northern Argentina (Peris and Mariluis 1984). Three subfamilies of Mesembrinellidae have been proposed (Guimarães 1977) and are widely accepted: Souzalopesiellinae and Laneellinae with a brown, non-metallic abdomen, and Mesembrinellinae with a metallic abdomen (Vargas and Wood 2012, Marinho et al. 2016). On the other hand, six subfamilies of Calliphoridae occur in the Neotropics: Calliphorinae, Chrysomyinae, Luciliinae, Melanomyinae, Polleniinae, and Toxotarsinae (Rognes 1991, 1997, Whitworth 2010).

Blow flies include more than 150 genera and approximately 1500 species worldwide (Rognes 1991, Pape et al. 2011). The adults of some species can impact human health, acting as vectors of pathogens by searching for and settling on feces, fresh and cooked meat, dairy products and wounds (Rognes 1991). The larvae of other species, e.g. *Cochliomyia hominivorax* (Coquerel, 1858), produce myiasis, invading and feeding on the tissues of live vertebrates, including humans (Zumpt 1965, Guimarães and Papavero 1999, Stevens et al. 2006). There are also blood-sucking species ectoparasitic on birds or mammals, e.g., *Protocalliphora* Hough, 1899 on nestling birds and *Auchmeromyia* Brauer & Bergenstamm, 1891 on humans (Rognes 1991). Blow flies are significant in forensic medicine because they are among the first insects to colonize animal remains (Smith 1986). Some species have been suggested as an effective tool for assessment of vertebrate biodiversity, representing an indirect source of DNA from the vertebrate carcasses on which they have fed (Calvignac-Spencer et al. 2013). They are also considered potential environmental indicators in tropical areas since many species, e.g., *Mesembrinella bellardiana* Aldrich, 1922, are non-synanthropic and therefore strongly related to natural habitats (Gadelha et al. 2009).

Many authors have contributed to reviewing the taxonomy of Neotropical Calliphoridae *sensu lato* (i.e., Shannon 1926, Aubertin 1933, Hall 1948, Mello 1961, 1962, 1967, James 1970, Guimarães 1977, Dear 1979, 1985, Mariluis and Peris 1984, Peris and Mariluis 1984, Peris 1990, 1992, Mariluis et al. 1994a, 1994b, Mello 1996, Peris et al. 1998, Mello 2003, Peris and González-Mora 2005). In more recent taxonomic studies from the region, Vargas and Wood (2012) provided a comprehensive review and key to Central American genera; Whitworth (2010) studied the species present in the West Indies, providing keys and reviewing some species, as well as describing a new one; the same author carried out a complete revision of the six species of *Calliphora* Robineau-Desvoidy, 1830 from the Neotropical Region (Whitworth 2012) and a revision of 23 species of the genus *Lucilia* Robineau-Desvoidy, 1830 found in the Neotropics, where he provided an identification key and described six new species (Whitworth 2014). The recent revisions of some genera of Mesembrinellidae, including descriptions of new species (Wolff et al. 2012, Wolff 2013, Wolff et al. 2013, 2014) and the first phylogenetic study of this family (Marinho et al. 2016), have been significant. There are also a list of valid blow fly names from the Americas south of Mexico provided by Kosmann et al. (2013) and a catalogue of Calliphoridae and Mesembrinellidae of Colombia (Wolff and Kosmann 2016).

Furthermore, lists of species, identification keys and ecological studies can be found for Nicaragua (Maes et al. 1994), Panama (Bermudez 2007), Colombia (Pape et al. 2004, Amat et al. 2008, Amat 2009), Brazil (Carvalho and Ribeiro 2000), Peru (Baumgartner and Greenberg 1983, 1984, 1985) and Argentina (Mariluis 1981, 1983, 2002, Mariluis and Mulieri 2003). Background information regarding blow flies in Venezuela is more limited. A first list of Venezuelan blow flies was published by Cova (1964). Other studies have focused on a few species that can cause myiasis (Moissant et al. 2004a, 2004b, Coronado and Kowalski 2009, Pulgar et al. 2009) and on forensically important species (Liria 2006, Magaña et al. 2006, Velásquez 2008, Vásquez and Liria 2012, Capote et al. 2014, Nuñez and Liria 2014).

In this paper, for the first time, a checklist is presented of valid species names of Calliphoridae and Mesembrinellidae so far known to occur in Venezuela, as well as distribution maps of each species in the country.

## Materials and methods

The checklist is based on the examination of adult blow flies deposited in Venezuela’s main entomological collections, combined with our own data and a detailed bibliographic review. We examined specimens housed in the following museums and institutions:

BMNH The Natural History Museum, London, United Kingdom

CEUA Colección Entomológica de la Universidad de Alicante, Alicante, Spain

IVIC Colecciones Biológicas del Instituto Venezolano de Investigaciones Científicas, Caracas, Venezuela

MIZA Museo del Instituto de Zoología Agrícola Francisco Fernández Yépez, Universidad Central de Venezuela, Maracay, Venezuela

MJMO Museo Entomológico “Dr. José Manuel Osorio”, Universidad Centroccidental Lisandro Alvarado, Barquisimeto, Venezuela

Some of the specimens deposited in CEUA and IVIC were collected by the authors using Wind Oriented Traps (WOT) baited with fish and pig liver (see Vogt et al. 1985). The classification used in the checklist follows Rognes (1986, 1991, 1997) and Marinho et al. (2016). The material examined was identified on the basis of specific keys for each subfamily of Calliphoridae, i.e. Mariluis and Peris (1984) and Whitworth (2012) for Calliphorinae; Mariluis and Peris (1984), Mariluis et al. (1994b), Rognes (1994) and Whitworth (2010, 2014) for Luciliinae; Dear (1985), González-Mora et al. (1998), Mariluis et al. (1994a), Rognes and Paterson (2005), Whitworth (2010) and Grella et al. (2015) for Chrysomyinae, and Dear (1979) for Toxotarsinae. In the case of Mesembrinellidae the keys of Guimarães (1977), Bonatto and Marinoni (2005) and Wolff et al. (2014) were used. The dissection and study of male terminalia were carried out following Whitworth (2006, 2010). The identity of all specimens was confirmed by Dr Terry Whitworth from Washington State University (USA).

Localities of occurrence of both the examined material and records taken from the literature were georeferenced using Google Earth (v7.1.5.1557). Distribution maps were created with ArcView GIS 10.2 (Environmental Systems Research Institute, Inc., USA). Each point plotted on the maps represents a locality of occurrence. Distributions of species do not follow any alphabetic or taxonomic criterion but are instead represented in such a way as to avoid, as far as possible, the overlapping of dots.

## Results

Table 1 lists a total of 39 species of blow flies for Venezuela, of which 25 are Calliphoridae and 14 Mesembrinellidae. We examined a total of 498 specimens, the subfamily Chrysomyinae being the most abundant (302), followed by the Luciliinae (166). From the material examined we identified 26 species, including one Calliphoridae and two Mesembrinellidae newly recorded for the country: *Chrysomya putoria* (Wiedemann, 1830), *Mesembrinella spicata* Aldrich, 1925 and *Mesembrinella umbrosa* Aldrich, 1922.

**Figure 1.**
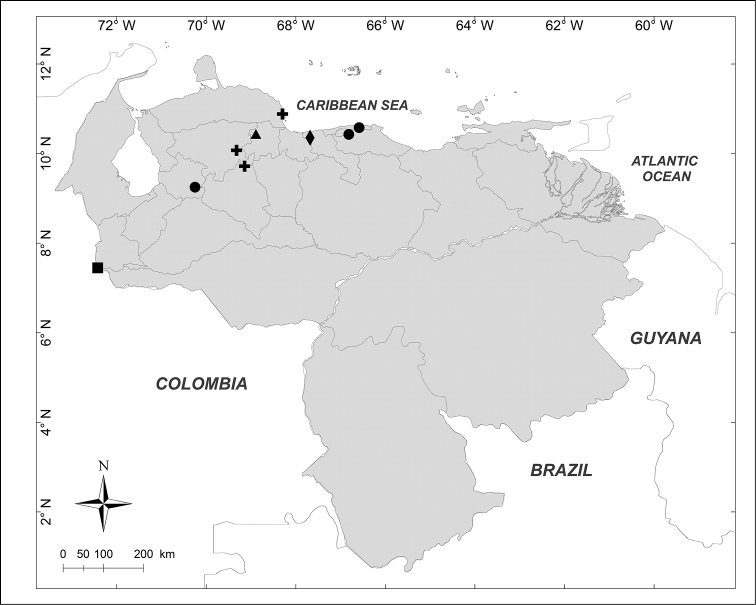
Known distributions of ■ *Calliphora nigribasis* Macquart, + *Cochliomyia hominivorax* (Coquerel), ● *Compsomyiops verena* (Walker), ▲ *Paralucilia fulvinota* (Bigot) and ◆ *Lucilia rognesi* Whitworth in Venezuela.

**Figure 2.**
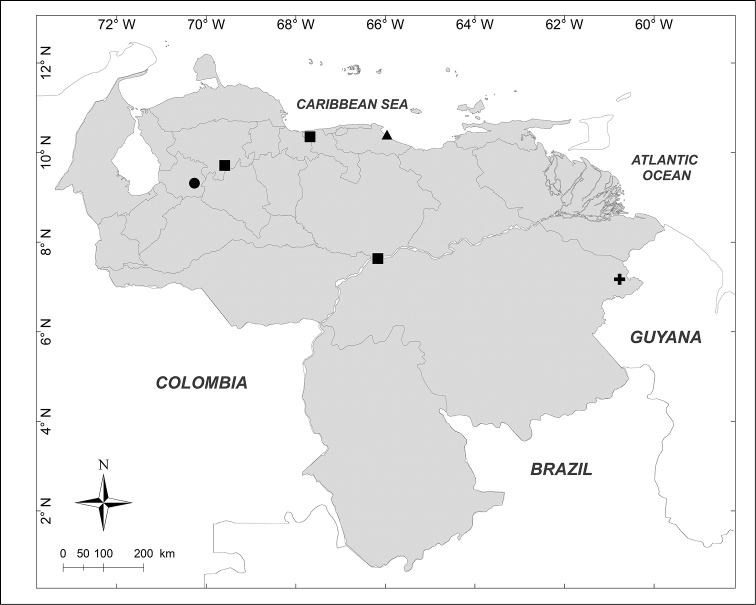
Known distributions of ■ *Mesembrinella bicolor* (Fabricius), ● *Mesembrinella umbrosa* Aldrich, + *Eumesembrinella benoisti* (Séguy) and ▲ *Thompsoniella anomala* Guimarães in Venezuela.

**Figure 3.**
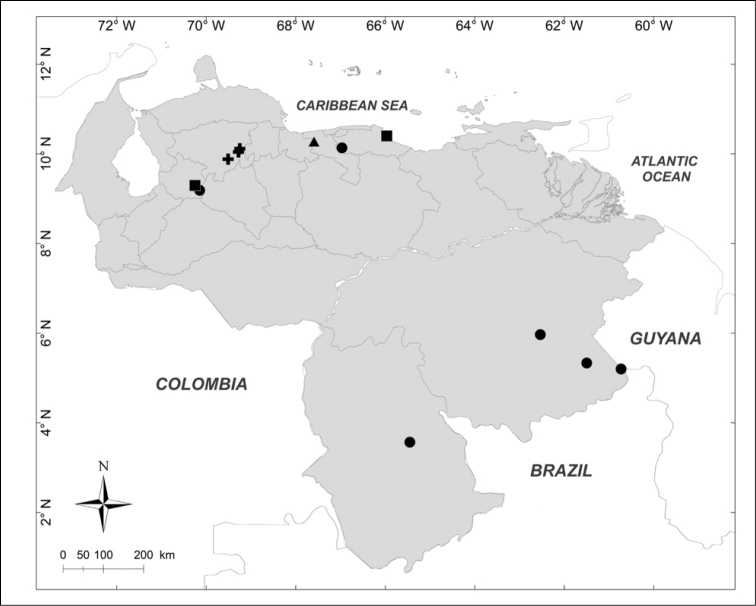
Known distributions of + *Chrysomya putoria* (Wiedemann), ■ *Souzalopesiella facialis* (Aldrich), ● *Sarconesia roraima* (Townsend) and ▲ *Chloroprocta idioidea* (Robineau-Desvoidy) in Venezuela.

**Figure 4.**
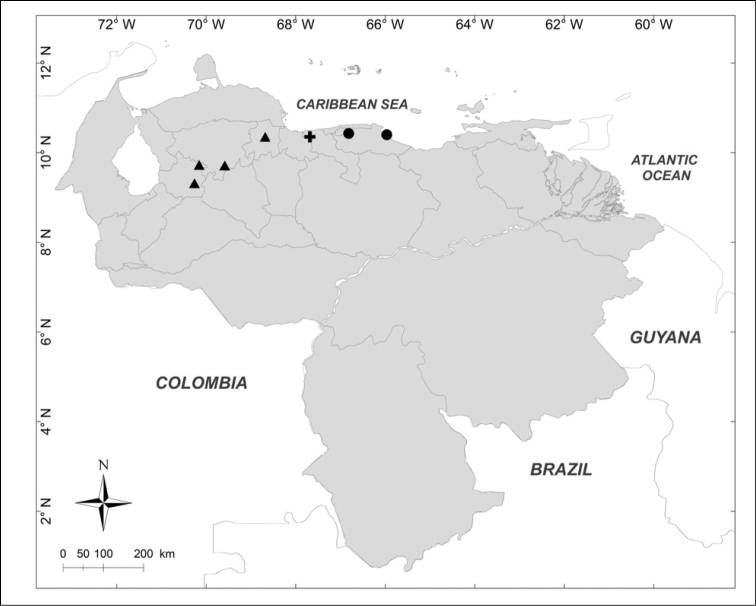
Known distributions of ▲ *Huascaromusca decrepita* (Séguy), ● *Huascaromusca lara* Bonatto, + *Lucilia nitida* Whitworth and ■ *Mesembrinella bellardiana* Aldrich in Venezuela.

**Figure 5.**
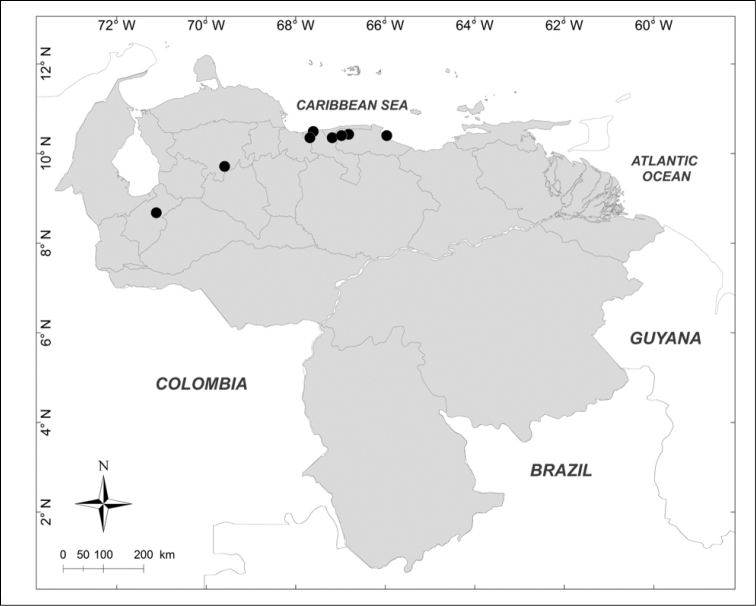
Known distributions of ● *Lucilia purpurascens* (Walker) in Venezuela.

**Figure 6.**
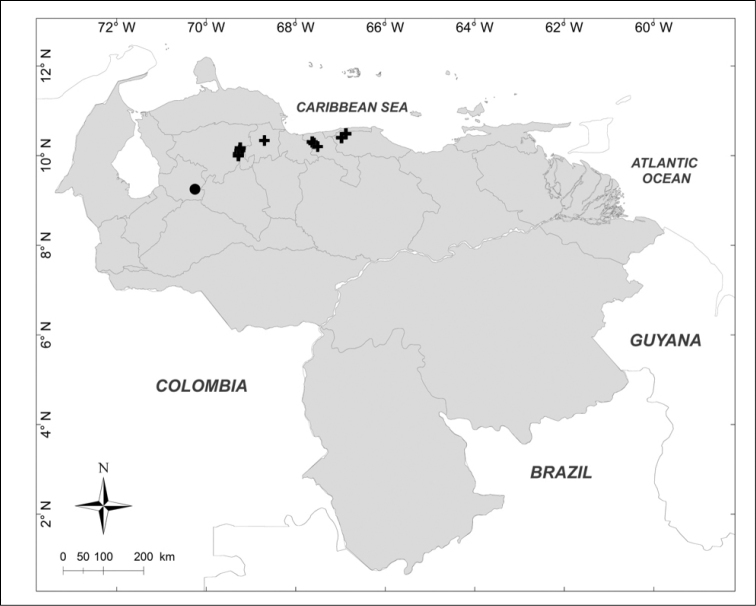
Known distributions of + *Lucilia eximia* (Wiedemann) and ● *Mesembrinella spicata* Aldrich in Venezuela.

**Figure 7.**
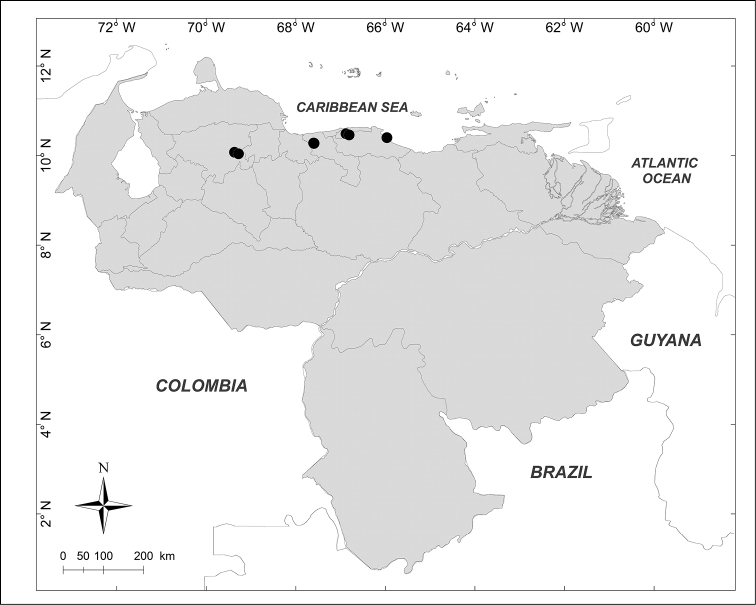
Known distributions of ● *Lucilia cuprina* (Wiedemann), ■ *Paralucilia paraensis* (Mello) and ▲ *Lucilia cluvia* (Walker) in Venezuela.

**Figure 8.**
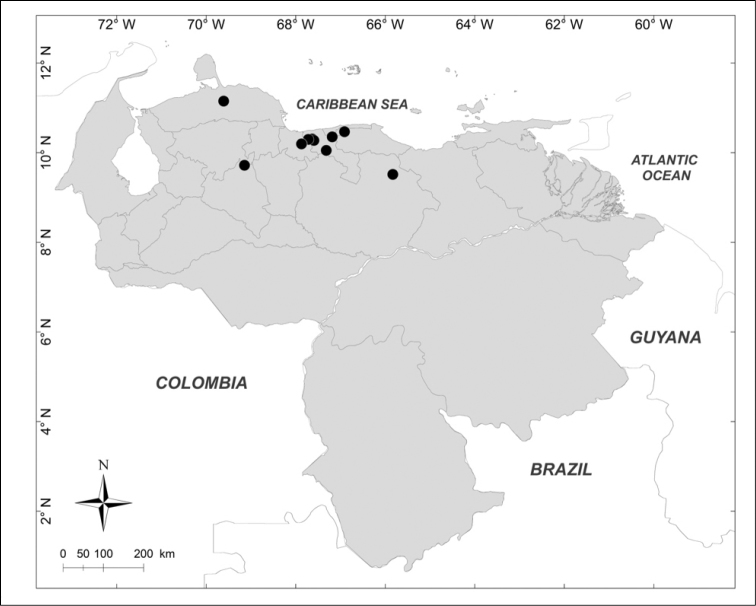
Known distribution of ● *Cochliomyia macellaria* (Fabricius) in Venezuela.

**Figure 9.**
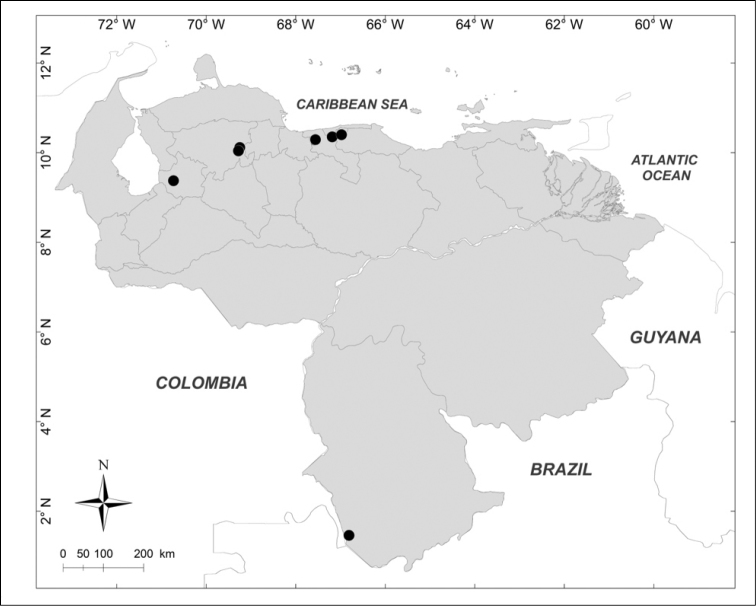
Known distribution of ● *Chrysomya albiceps* (Wiedemann) in Venezuela.

**Figure 10.**
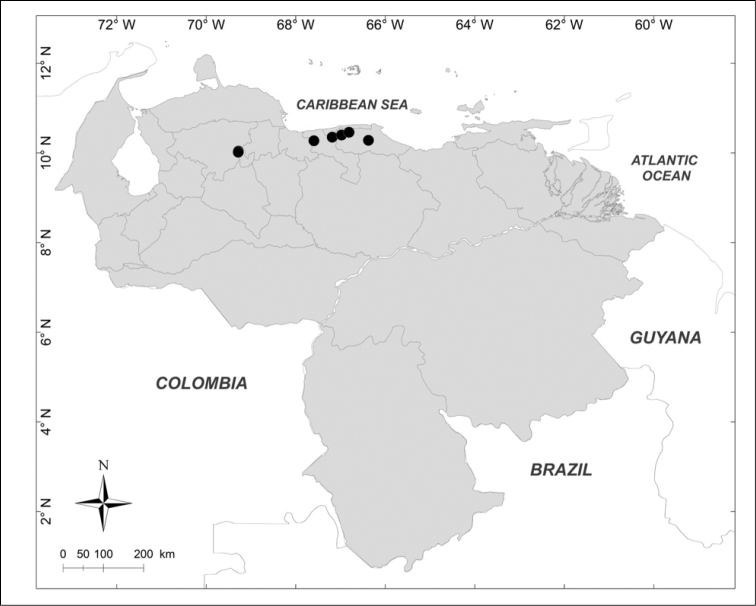
Known distribution of ● *Chrysomya megacephala* (Fabricius) in Venezuela.

**Figure 11.**
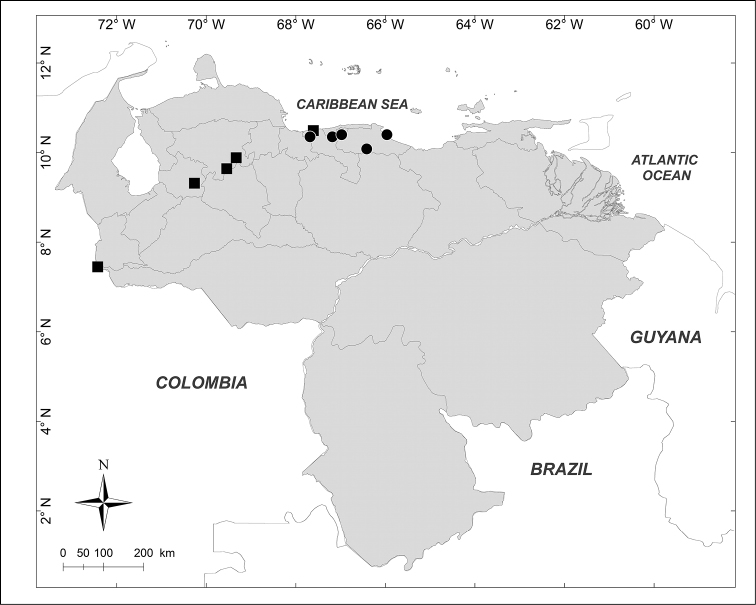
Known distributions of ■ *Blepharicnema splendens* Macquart and ● *Hemilucilia semidiaphana* (Rondani) in Venezuela.

**Figure 12.**
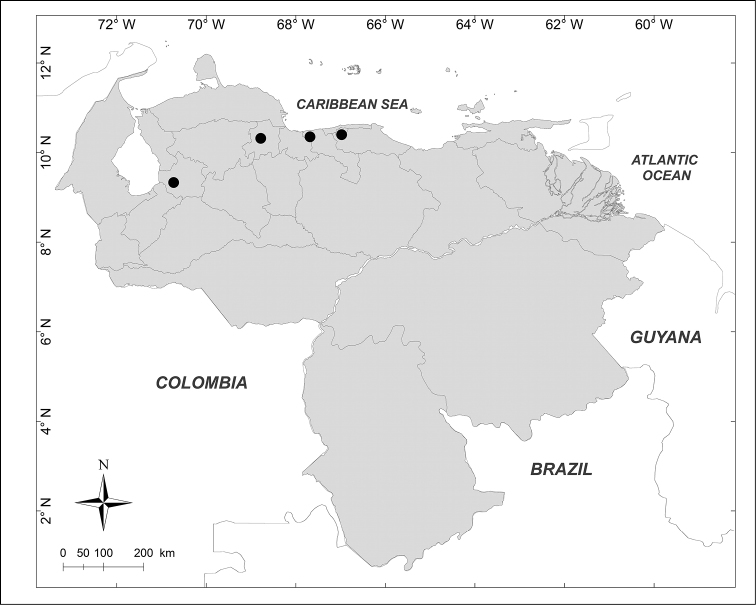
Known distribution of ● *Hemilucilia segmentaria* (Fabricius) in Venezuela.

**Figure 13.**
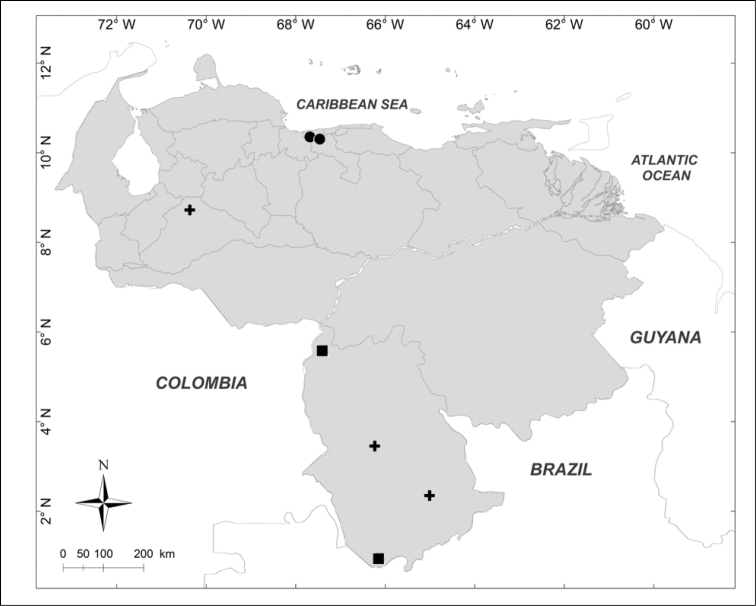
Known distributions of ● *Compsomyiops fulvicrura* (Robineau-Desvoidy), ■ *Lucilia albofusca* Whitworth and + *Hemilucilia benoisti* (Séguy) in Venezuela

**Figure 14.**
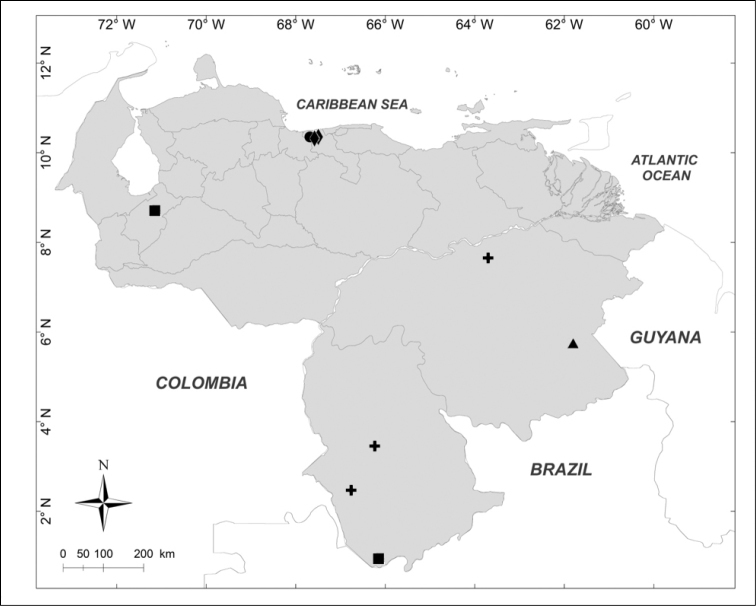
Known distributions of ● *Huascaromusca vogelsangi* Mello, ■ *Lucilia vulgata* Whitworth, ▲ *Giovanella bolivar* Bonatto, + *Eumesembrinella quadrilineata* (Fabricius) and ◆ *Mesembrinella xanthorrhina* (Bigot) in Venezuela.

Doubtful records found in the literature were excluded from the list when there was no indication of how the species were identified or when the accuracy of the identifications was uncertain. Distribution maps showing the records obtained from the material examined and the literature are provided for 37 species (Figs 1–14). *Eumesembrinella randa* (Walker, 1849) and *Lucilia sericata* (Meigen, 1826) were cited for Venezuela by Peris and Mariluis (1984), Mariluis et al. (1994b), Kosmann et al. (2013) and Wolff and Kosmann (2016), but no locality information was provided.

**Table 1.** Checklist of the blow flies of Venezuela, including reviewed references and the depositories of examined specimens.

Species	References	Material examined
**FAMILY CALLIPHORIDAE**		
**Subfamily CALLIPHORINAE**		
*Calliphora nigribasis* Macquart, 1851	Cova (1964), Whitworth (2012), Kosmann et al. (2013), Wolff and Kosmann (2016)	MIZA
**Subfamily CHRYSOMYINAE**		
*Chloroprocta idioidea* (Robineau-Desvoidy, 1830)	Hall (1948), Cova (1964), Dear (1985), Kosmann et al. (2013), Wolff and Kosmann (2016)	BMNH
*Chrysomya albiceps* (Wiedemann, 1819)	Baumgartner (1988), Kosmann et al. (2013), Wolff and Kosmann (2016)	CEUA, IVIC, MJMO, MIZA
*Chrysomya megacephala* (Fabricius, 1794)	Baumgartner (1988)	CEUA, IVIC, MJMO
*Chrysomya putoria* (Wiedemann, 1830)	New record	MJMO
*Cochliomyia hominivorax* (Coquerel, 1858)	Moissant et al. (2004a, 2004b), Coronado and Kowalski (2009), Pulgar (2009)	MJMO
*Cochliomyia macellaria* (Fabricius, 1775)	Cova (1964), Dear (1985), Kosmann et al. (2013), Wolff and Kosmann (2016)	CEUA, IVIC, MIZA, MJMO
*Compsomyiops fulvicrura* (Robineau-Desvoidy, 1830)	Hall (1948), Cova (1964)	-
*Compsomyiops verena* (Walker, 1849)	Dear (1985), Kosmann et al. (2013), Wolff and Kosmann (2016)	MIZA
*Hemilucilia benoisti* Séguy, 1925a	Shannon (1926), Dear (1985), Peris and Mariluis (1989), Kosmann et al. (2013), Wolff and Kosmann (2016)	-
*Hemilucilia segmentaria* (Fabricius, 1805)	Shannon (1926), Hall (1948), Cova (1964), Peris and Mariluis (1989)	CEUA, IVIC, MIZA, MJMO
*Hemilucilia semidiaphana* (Rondani, 1850)	Dear (1985), Cova (1964), Peris and Mariluis (1989), Kosmann et al. (2013), Wolff and Kosmann (2016)	CEUA, IVIC, MIZA
*Paralucilia fulvinota* (Bigot, 1877)	Aldrich (1925), Shannon (1926), Dear (1985), Mariluis et al. (1994a), Kosmann et al. (2013), Wolff and Kosmann (2016)	MIZA
*Paralucilia paraensis* (Mello, 1969)	Dear (1985), Mariluis et al. (1994a), Kosmann et al. (2013), Wolff and Kosmann (2016)	-
**Subfamily LUCILIINAE**		
*Blepharicnema splendens* Macquart, 1843	Cova (1964), Mariluis and Peris (1984), Amat and Wolff (2007), Kosmann et al. (2013), Wolff and Kosmann (2016)	MIZA, MJMO
*Lucilia albofusca* Whitworth, 2014	Whitworth (2014)	-
*Lucilia cluvia* (Walker, 1849)	Mariluis et al. (1994b)	-
*Lucilia cuprina* (Wiedemann, 1830)	Cova (1964), Kosmann et al. (2013), Wolff and Kosmann (2016)	CEUA, IVIC, MIZA, MJMO
*Lucilia eximia* (Wiedemann, 1819)	Cova (1964), Mariluis et al. (1994b), Kosmann et al. (2013), Whitworth (2014), Wolff and Kosmann (2016)	IVIC, MIZA, MJMO
*Lucilia nitida* Whitworth, 2014	Whitworth (2014)	CEUA
*Lucilia purpurascens* (Walker, 1836)	Cova (1964), Mariluis et al. (1994b), Kosmann et al. (2013), Whitworth (2014), Wolff and Kosmann (2016)	CEUA, MIZA, MJMO, IVIC
*Lucilia rognesi* Whitworth, 2014	Whitworth (2014)	CEUA
*Lucilia sericata* (Meigen, 1826)	Mariluis et al. (1994b), Kosmann et al. (2013), Wolff and Kosmann (2016)	-
*Lucilia vulgata* Whitworth, 2014	Whitworth (2014)	-
**Subfamily TOXOTARSINAE**		
*Sarconesia roraima* (Townsend, 1935)	Dear (1979), Mariluis and Peris (1984), Wolff and Kosmann (2016)	MIZA
**FAMILY MESEMBRINELLIDAE**		
*Eumesembrinella benoisti* (Séguy, 1925b)	Guimarães (1977), Kosmann et al. (2013), Wolff and Kosmann (2016)	MIZA
*Eumesembrinella quadrilineata* (Fabricius, 1805)	Aldrich (1922), Guimarães (1977), Peris and Mariluis (1984), Kosmann et al. (2013), Wolff and Kosmann (2016)	-
*Eumesembrinella randa* (Walker, 1849)	Peris and Mariluis (1984), Kosmann et al. (2013), Wolff and Kosmann (2016)	-
*Giovanella bolivar* Bonatto, 2005	Bonatto and Marinoni (2005), Kosmann et al. (2013)	-
*Huascaromusca decrepita* (Séguy, 1925b)	Kosmann et al. (2013), Wolff and Kosmann (2016)	CEUA, MIZA, MJMO
*Huascaromusca lara* Bonatto, 2005	Bonatto and Marinoni (2005), Kosmann et al. (2013)	IVIC, MIZA
*Huascaromusca vogelsangi* Mello, 1967	Guimarães (1977), Kosmann et al. (2013), Wolff and Kosmann (2016)	-
*Mesembrinella bellardiana* Aldrich, 1922	Peris and Mariluis (1984), Kosmann et al. (2013), Wolff and Kosmann (2016)	-
*Mesembrinella bicolor* (Fabricius, 1805)	Aldrich (1922), Guimarães (1977), Peris and Mariluis (1984)	MIZA, MJMO
*Mesembrinella spicata* Aldrich, 1925	New record	MJMO
*Mesembrinella umbrosa* Aldrich, 1922	New record	MJMO
*Mesembrinella xanthorrhina* (Bigot, 1887)	Hall (1948), Cova (1964)	-
*Souzalopesiella facialis* (Aldrich, 1922)	Guimarães (1977), Kosmann et al. (2013), Wolff and Kosmann (2016)	CEUA, MJMO
*Thompsoniella anomala* Guimarães, 1977	Guimarães (1977), Kosmann et al. (2013)	CEUA

### Material examined

#### Family Calliphoridae

##### Subfamily Calliphorinae

***Calliphora nigribasis* Macquart, 1851** (Fig. 1)

Material examined (1 male): **Táchira State**: Betania, 2325m, 7.VIII.1972, J.B. Terán J. Salcedo leg. (MIZA).

##### Subfamily Chrysomyinae

***Chloroprocta idioidea* (Robineau-Desvoidy, 1830)** (Fig. 3)

Material examined (1 male, 1 female): **Aragua State**: Maracay, 29.VIII.1943, [no collector] (BMNH).

***Chrysomya albiceps* (Wiedemann, 1819)** (Fig. 9)

Material examined (38 males, 78 females): **Aragua State**: 2 males, Parque Nacional Henri Pittier, Portachuelo, 1152m, 26.I.2007, A. Martínez-Sánchez leg. (CEUA); 21 males, 57 females, Maracay, Universidad Central de Venezuela campus, 10°16'24.83"N, 67°35'37.05"W, approx. 400m, on dead chicken, various dates: 1 male, 10 females, 17.VII.2012; 1 male, 4 females, 18.VII.2012; 1 male, 1 female, 19.VII.2012; 1 male, 5 females, 20.VII.2012; 5 males, 36 females, 23.VII.2012; 12 males, 1 female, 27.VII.2012; all A. Thomas leg. (IVIC); 8 females, Maracay, Universidad Central de Venezuela campus, 24.I.2007, A. Martínez-Sánchez leg. (CEUA). **Lara State**: 5 males, 3 females, El Cercado, 500m, 25.VII.2002, from larva in dead common opossum, E. Arcaya leg. (MJMO); 9 males, Tarabana, 500m, XII.1989, A. Chávez leg. (MJMO). **Miranda State**: 1 male, 4 females, Altos de Pipe, Instituto Venezolano de Investigaciones Científicas, 10°24'5"N, 66°58'37"W, 1600m, 29.VII–2.VIII.2010, on dead rat, A. Thomas leg. (IVIC); 5 females, Macaracuay, Residencia Los Cien, 10°27'43.47"N, 66°48'34.71"W, 900m, on mango, 4.IX.2012, A. Thomas leg. (IVIC). **Trujillo State**: 1 female, La Cira, nr Betijoque, 500m, 4–9.XII.1996, J. Clavijo, J. de Marmels, J.L. García, A. Chacón leg. (MIZA).

***Chrysomya megacephala* (Fabricius, 1794)** (Fig. 10)

Material examined (60 males, 162 females): **Aragua State**: 1 female, Parque Nacional Henri Pittier, Portachuelo, 1152m, 26.I.2007, A. Martínez-Sánchez leg. (CEUA); 3 females, Maracay, Universidad Central de Venezuela campus, 7.IX.2006, from larva in chicken, students leg. (IVIC); 48 males, 138 females, Maracay, Universidad Central de Venezuela campus, 10°16'24.83"N, 67°35'37.05"W, approx. 400m, on dead chicken, various dates: 15 males, 81 females, 17.VII.2012; 4 males, 11 females, 18.VII.2012; 4 males, 13 females, 19.VII.2012; 4 males, 13 females, 23.VII.2012; 21 males, 20 females, 27.VII.2012; all A. Thomas leg. (IVIC); 1 male, 10 females, Maracay, Universidad Central de Venezuela campus, 24.I.2007, A. Martínez-Sánchez leg. (CEUA). **Lara State**: 1 male, 1 female, Barquisimeto, Museo Entomológico “Dr. José Manuel Osorio”, 564m, VI.1989, on trunk of Acacia plagued by scale insect, [no collector] (MJMO); 2 males, 2 females, Tarabana, 500m, VI.1989, Acht leg. (MJMO); 1 female, 21.XII.1993–10.I.1994, Malaise trap [no collector] (MJMO). **Miranda State**: 1 male, Caucagua, 74m, 18–20.VII.2000, E. Carrasquero leg. (MJMO); 1 male, 1 female, Altos de Pipe, Instituto Venezolano de Investigaciones Científicas, 10°24'5"N, 66°58'37"W, 1600m, 29.VII–2.VIII.2010, on dead rat, A. Thomas leg. (IVIC); 6 males, 5 females, Macaracuay, Residencia Los Cien, 10°27'43.47"N, 66°48'34.71"W, 900m, 4.IX.2012, on mango, A. Thomas leg. (IVIC).

***Chrysomya putoria* (Wiedemann, 1830)** (Fig. 3)

Material examined (2 males, 3 females): **Lara State**: 1 female, El Cercado, 500m 17.XII.1996, E. Arcaya leg. (MJMO); 1 male, San Miguel, 680m, 17.VI.1993, H. Chávez, R. Hernández leg. (MJMO); 1 male, 2 females, Tarabana, XII.1989, A. Chavez leg. (MJMO).

***Cochliomyia hominivorax* (Coquerel, 1858)** (Fig. 1)

Material examined (2 males, 2 females): **Falcón State**: 1 female, Parque Nacional Morrocoy, 20.III–IV.1999, H. Chávez leg. (MJMO). **Lara State**: 1 male, Barquisimeto, 564m, VI.1980, myiasis in *Canis familiaris*, C. Zambrano leg. (MJMO); 1 male, 1 female, Sanare, El Torrellero, 268m, 20.IV.1982, Malaise trap, [no collector] (MJMO).

***Cochliomyia macellaria* (Fabricius, 1775)** (Fig. 8)

Material examined (3 males, 6 females): **Aragua State**: 1 male, El Limón, 450m, 22.II.1973, J.C. Marín leg. (MIZA); 1 female, Parque Nacional Henri Pittier, Portachuelo, 1152m, 26.I.2007, A. Martínez-Sánchez leg. (CEUA); 1 female, Maracay, Universidad Central de Venezuela campus, 7.IX.2006, students leg. (IVIC); 1 male, Villa del Cura, Estación Experimental Cataurito, 1000m, 9.IV.1981, J.L. García leg. (MIZA). **Carabobo State**: 1 female, Mariara, 12.VII.1979, F. Alarcón leg. (MIZA). **Falcón State**: 1 female, Cabure, 7.VI.1980, light trap, R. Casales, E. Zambrano leg. (MIZA). **Guárico State**: 1 female, Distrito Rivas, Carretera El Palmar km 133, La Smith, 4.VIII.1980, J. Valdivieso leg. (MIZA). **Lara State**: 1 female, Sanare, El Torrellero, 268m, 20.IV.1980, Malaise trap, [no collector] (MJMO). **Miranda State**: 1 male, Distrito Federal, El Valle, 10.XI.1949, on trunk of Bucare, F. Fernández Yépez leg. (MIZA).

***Compsomyiops verena* (Walker, 1849)** (Fig. 1)

Material examined (1 male, 2 females): **Miranda State**: 1 female, Distrito Federal, Serranía El Avila, Los Castillitos, 1300m, 24.III.1950, F. Fernández Yépez leg. (MIZA); 1 female, El Hatillo, Las Marías, 1350m, 5.II.1976, F. Kaletta leg. (MIZA). **Trujillo State**: 1 male, Carretera Boconó, La Negrita, 1850m, 29.X.1976, J. Salcedo & J. Clavijo leg. (MIZA).

***Hemilucilia segmentaria* (Fabricius, 1805)** (Fig. 12)

Material examined (2 males, 3 females): **Aragua State**: 1 female, Parque Nacional Henri Pittier, Rancho Grande, 1183m, 25.I.2007, A. Martínez-Sánchez leg. (CEUA). **Miranda State**: 1 male, Altos de Pipe, Instituto Venezolano de Investigaciones Científicas, 10°24'5"N, 66°58'37"W, 1600m, 7.II.2012, A. Thomas leg. (IVIC). **Trujillo State**: 1 male, 1 female, La Gira, nr Betijoque, 500m, 4–9.XII.1996, J. Clavijo, J. de Marmels, J.L. García, A. Chacón leg. (MIZA). **Yaracuy State**: 1 female, Cocorote, Sector El Candelo, 1650m, 17–20.X.2001, interception trap, R. Briceño, A. Chacón, J. Clavijo, F. Díaz, R. Paz, E. Arcaya, L. Joly leg. (MJMO).

***Hemilucilia semidiaphana* (Rondani, 1850)** (Fig. 11)

Material examined (5 males, 48 females): **Aragua State**: 4 males, 7 females, Parque Nacional Henri Pittier, Portachuelo, 1152m, 26.I.2007, A. Martínez-Sánchez leg. (CEUA); 13 females, Parque Nacional Henri Pittier, Rancho Grande, 1183m, 24–25.I.2007, WOT, A. Martínez-Sánchez leg. (CEUA); 19 females, 1183m, 25.I.2007, WOT, A. Martínez-Sánchez leg. (CEUA); 1 female, 1100m, 17.V.1973, J. Salcedo, J. Clavijo leg. (MIZA). **Miranda State**: 1 male, Guatopo (Agua Blanca), 8.X.1980, F. Fernández Yépez, A. Chacón leg. (MIZA); 1 female, San Antonio de los Altos, Instituto Venezolano de Investigaciones Científicas, 1680m, IV.2003, Y. Velásquez leg. (IVIC); 7 females, Altos de Pipe, Instituto Venezolano de Investigaciones Científicas, 10°24'5"N 66°58'37"W, 1600m, 7.II.2012, A. Thomas leg. (IVIC).

***Paralucilia fulvinota* (Bigot, 1877)** (Fig. 1)

Material examined (1 male): **Yaracuy State**: Aroa, 12.VIII.1975, E. Dietz leg. (MIZA).

##### Subfamily Luciliinae

***Blepharicnema splendens* Macquart, 1843** (Fig. 11)

Material examined (4 males, 3 females): **Aragua State**: 1 female, Choroní, 1600m, 4.XI.1971, C.J. Rosales leg. (MIZA). **Lara State**: 2 males, Parque Nacional Yacambú, 15.X.1982, F. Gutiérrez, F. Martínez leg. (MIZA); 2 males, Piedra del Tigre, 1300m, 19.XI.2002, F. Díaz, F. Sosa, N. Valera leg. (MJMO). **Táchira State**: 1 female, Betania, on the route to the Páramo El Tamá, 2425m, 16–20.III.1983, “Excursión Instituto de Zoología Agrícola” leg. (MIZA). **Trujillo State**: 1 female, Parque Nacional Guaramacal, 1480m, 11–16.VI.2002, yellow pan trap, R. Briceño, J. Clavijo, R. Paz, F. Díaz, L. Joly, A. Chacón leg. (MJMO).

***Lucilia cuprina* (Wiedemann, 1830)** (Fig. 7)

Material examined (5 males, 11 females): **Aragua State**: 1 male, 4 females, Maracay, Universidad Central de Venezuela campus, 10°16'24.83"N, 67°35'37.05"W, approx. 400m, on dead chicken, various dates: 1 male, 2 females, 17.VII.2012; 2 females, 17–26.VII.2012; all A. Thomas leg. (IVIC). **Lara State**: 3 females, Los Crespúsculos, 500m, 16.VII.1999, J. Nieto leg. (MJMO); 1 female, Tarabana, 500m, V.1990, myiasis on *Canis familiaris*, C. Zambrano leg. (MJMO). **Miranda State**: 1 female, Distrito Federal, Caracas, 1.II.1974, from larva on dead fish, F. Kaletta leg. (MIZA); 1 female, San Antonio de los Altos, Instituto Venezolano de Investigaciones Científicas, 1680m, 22.I.2007, A. Martínez-Sánchez leg. (CEUA); 4 males, 1 female, Macaracuay, Residencia Los Cien, 10°27'43.47"N, 66°48'34.71"W, 900m, 29.VII.2012, on mango, A. Thomas leg. (IVIC).

***Lucilia eximia* (Wiedemann, 1819)** (Fig. 6)

Material examined (19 males, 15 females): **Aragua State**: 1 female, El Limón, 480m, 27.V.1973, Malaise trap, C.J. Rosales leg. (MIZA); 1 female, Maracay, Universidad Central de Venezuela campus, 6.IX.2006, students leg. (IVIC). **Lara State**: 2 females, Cordero, 600m, 27–30.VI.1992, interception trap, [no collector] (MJMO); 4 males, 1 female, El Cercado, 500m, Malaise trap, various dates: 1 male, 17–21.VI.1999; 1 male, 1 female, 24.VI–5.VII.1999; 2 males, 5–11.VII.1999; [all no collector] (MJMO); 5 males, El Cercado, 500m, 13.V.2002, on dead fish, E. Arcaya leg. (MJMO); 1 male, El Cercado, V.2002, [no collector] (MJMO); 2 males, 2 females, La Mora, 400m, 17.VI.2012, on *Stapelia gigantea*, T. Capote leg. (MJMO); 5 males, 3 females, Tarabana, 500m, various dates: 3 males, 1 female, 1.VI.2002; 1 male, 1 female, VII.2002; 1 male, 1 female, 14.II.2003; all E. Arcaya leg. (MJMO); 1 male, Tarabana, 17.VI.2002, on liver bait, E. Arcaya leg. (MJMO). **Miranda State**: 3 females, Altos de Pipe, Instituto Venezolano de Investigaciones Científicas, 10°24'5"N, 66°58'37"W, 1600m, 29.VII–2.VIII.2010, on dead rat, A. Thomas leg. (IVIC); 1 male, 1 female, Distrito Federal, Caracas, Jardín Botánico, 9.III.1966, A. Díaz leg. (MIZA). **Yaracuy State**: 1 male, 1 female, Cocorote, Sector El Candelo, 1600m, 4–10.XI.2002, R. Briceño, A. Chacón, J. Clavijo, F. Díaz, R. Paz, E. Arcaya, L. Joly leg. (MJMO).

***Lucilia nitida* Whitworth, 2014** (Fig. 4)

Material examined (2 females): **Aragua State**: Parque Nacional Henri Pittier, Rancho Grande, 1183m, 25.I.2007, WOT, A. Martínez-Sánchez leg. (CEUA).

***Lucilia purpurascens* (Walker, 1837)** (Fig. 5)

Material examined (13 males, 58 females): **Aragua State**: 1 female, Choroní, 1400m, 10.X.1952, F. Kern leg. (MIZA); 4 females, Parque Nacional Henri Pittier, Portachuelo, 1152m, 26.I.2007, A. Martínez-Sánchez leg. (CEUA); 1 female, Parque Nacional Henri Pittier, Rancho Grande, 1100m, 30.IX.1974, J.L. García leg. (MIZA); 1 female, Parque Nacional Henri Pittier, Rancho Grande, 1183m, 25.I.2007, A. Martínez-Sánchez leg. (CEUA). **Lara State**: 1 female, Parque Nacional Yacambú, El Blanquito, 1480m, 14–21.IX.2001, Malaise trap, R. Briceño, A. Chacón, J. Clavijo, F. Díaz, R. Paz, E. Arcaya leg. (MJMO); 1 female, Parque Nacional Yacambú, El Blanquito, 11–16.III.2002, yellow pan trap, R. Briceño, J. Clavijo, F. Díaz, R. Paz, E. Arcaya, A. Chacón leg. (MJMO). **Mérida State**: 1 female, Mérida, Hotel Valle Grande, 2000m, 2.IX.1980, C.J. Rosales leg. (MIZA). **Miranda State**: 1 male, El Hatillo, Las Marías, 1350m, 16.VI.1975, F. Kaletta leg. (MIZA); 12 males, 40 females, San Antonio de los Altos, Instituto Venezolano de Investigaciones Científicas, 1680m, 13.II.2007, reared from larva, mother collected on fish, A. Martínez-Sánchez leg. (CEUA); 2 females, Altos de Pipe, Instituto Venezolano de Investigaciones Científicas, 10°24'5"N, 66°58'37"W, 1600m, 29.VII–2.VIII.2010, on dead rat, A. Thomas leg. (IVIC); 2 females, Altos de Pipe, Instituto Venezolano de Investigaciones Científicas, 10°24'5"N, 66°58'37"W, 1600m, 7.II.2012, A. Thomas leg. (IVIC). **Yaracuy State**: 4 females, Cocorote, Sector El Candelo, 1650m, 17–20.X.2001, interception trap, R. Briceño, A. Chacón, J. Clavijo, F. Díaz, R. Paz, E. Arcaya, L. Joly leg. (MJMO).

***Lucilia rognesi* Whitworth, 2014** (Fig. 1)

Material examined (2 females): **Aragua State**: Parque Nacional Henri Pittier, Rancho Grande, 1183m, 25.I.2007, WOT, A. Martínez-Sánchez leg. (CEUA).

Subfamily Toxotarsinae

***Sarconesia roraima* (Townsend, 1935)** (Fig. 3)

Material examined (5 males, 3 females): **Amazonas State**: 1 male, Parque Nacional Duida, Cerro Marahuaka, 2470m, 3–6.XI.1992, “Expedición Terramar”, J. Clavijo, A. Chacón leg. (MIZA). **Bolívar State**: 1 female, Auyentepui, 2150m, 26.II.1978, L. Joly leg. (MIZA); 1 male, Gran Sabana, Cerro Kukenan, 2700m, 12–17.IV.1988, A. Chacón, C. Andara leg. (MIZA); 1 female, Gran Sabana, Cerro Roraima, 2700m, 12–21.I.1991, “Expedición Terramar”, A. Chacón leg. (MIZA). **Miranda State**: 2 males, 1 female, Distrito Federal, El Junquito, Estación Experimental Bajo Seco, 1900m, 17.IV.1976, C.J. Rosales leg. (MIZA). **Trujillo State**: 1 male, Carretera Boconó, Guaramacal, 2300m, 29.X.1976, C.J. Rosales, J.L. García leg. (MIZA).

#### Family Mesembrinellidae

***Eumesembrinella benoisti* (Séguy, 1925b)** (Fig. 2)

Material examined (1 female): **Bolívar State**: Reserva Forestal Imataca, El Bochinche, 200m, 6–18.XII.1974, “Expedición IZT– UCV” leg. (MIZA).

***Huascaromusca decrepita* (Séguy, 1925b)** (Fig. 4)

Material examined (6 males, 1 female): **Lara State**: 1 male, 1 female, Parque Nacional Yacambú, El Blanquito, 29.I.2007, A. Martínez-Sánchez leg. (CEUA). **Trujillo State**: 2 males, San Isidro, 14 km Sur, La Soledad, 1500m, 30–31.V.1975, Malaise trap, R.E. Dietz leg. (MIZA); 2 males, Parque Nacional Guaramacal, 1480m, 19–25.V.2001, yellow pan trap, R. Briceño, A. Chacón, J. Clavijo, F. Díaz, R. Paz leg. (MJMO). **Yaracuy State**: 1 male, Cocorote, Sector El Candelo, 1650m, 17–20.X.2001, R. Briceño, A. Chacón, J. Clavijo, F. Díaz, R. Paz, E. Arcaya, L. Joly leg. (MJMO).

***Huascaromusca lara* Bonatto *in* Bonatto & Marinoni, 2005** (Fig. 4)

Material examined (2 females): **Miranda State**: 1 female, El Hatillo, Las Marías, 1350m, 26.V.1976, F. Kaletta leg. (MIZA); 1 female, San Antonio de los Altos, Instituto Venezolano de Investigaciones Científicas, 1680m, IV.2003, Y. Velásquez leg. (IVIC).

***Mesembrinella bicolor* (Fabricius, 1805)** (Fig. 2)

Material examined (3 males): **Aragua State**: 1 male, Parque Nacional Henri Pittier, Rancho Grande, 1100m, 1.VI.1981, A. Field leg. (MIZA). **Bolívar State**: 1 male, Carretera Caicara, San Juan de Manapiare, 300m, 21–30.VII.1973, J.L. García leg. (MIZA). **Lara State**: 1 male, El Blanquito, 1480m, 11–16.III.2002, R. Briceño, A. Chacón, J. Clavijo, F. Díaz, R. Paz, L. Joly leg. (MJMO).

***Mesembrinella spicata* Aldrich, 1925** (Fig. 6)

Material examined (2 females): **Trujillo State**: Parque Nacional Guaramacal, 1480m, 14–20.II.2002, R. Briceño, A. Chacón, J. Clavijo, F. Díaz, R. Paz, L. Joly leg. (MJMO).

***Mesembrinella umbrosa* Aldrich, 1922** (Fig. 2)

Material examined (1 male): **Trujillo State**: Parque Nacional Guaramacal, 1480m, 14–20.II.2002, R. Briceño, A. Chacón, J. Clavijo, F. Díaz, R. Paz, L. Joly leg. (MJMO).

***Souzalopesiella facialis* (Aldrich, 1922)** (Fig. 3)

Material examined (3 males, 1 female): **Aragua State**: 1 female, Parque Nacional Henri Pittier, Rancho Grande, 1183m, 25.I.2007, A. Martínez-Sánchez leg. (CEUA). **Lara State**: 1 male, Parque Nacional Yacambú, El Blanquito, 1480m, 11–16.III.2002, R. Briceño, J. Clavijo, R. Paz, F. Díaz, E. Arcaya, A. Chacón leg. (MJMO). **Trujillo State**: 2 males, Parque Nacional Guaramacal, 1480m, 14–20.II.2002, yellow pan trap, R. Briceño, A. Chacón, J. Clavijo, F. Díaz, R. Paz, L. Joly leg. (MJMO).

***Thompsoniella anomala* Guimarães, 1977** (Fig. 2)

Material examined (1 female): **Miranda State**: San Antonio de los Altos, Instituto Venezolano de Investigaciones Científicas, 1680m, 22.I.2007, A. Martínez-Sánchez leg. (CEUA).

## Discussion

This study is the first to determine the diversity of Calliphoridae and Mesembrinellidae in Venezuela. The checklist contains a total of 39 species of Calliphoridae, with 25 species distributed in ten genera, and Mesembrinellidae, with 14 species distributed in six genera. Twenty-six species were identified from examined material, while 13 species are listed based exclusively on records found in the literature. Compared to neighbouring countries that have been relatively well-studied, the known Venezuelan blow fly fauna is equivalent to that of Brazil (39 species: 24 Calliphoridae in eight genera and 15 Mesembrinellidae in seven genera) (Kosmann et al. 2013), but less diverse than that of Colombia (52 species: 31 Calliphoridae in 12 genera and 21 Mesembrinellidae in seven genera) (Wolff and Kosmann 2016).

Three species are newly recorded for the country: *Chrysomya putoria*, *Mesembrinella spicata* and *Mesembrinella umbrosa*. These records were not unexpected, as these species have been found in other South American countries: *Chrysomya putoria* in Argentina, Bolivia, Brazil, Colombia, Ecuador, Paraguay and Peru (Baumgartner 1988, Wolff and Kosmann 2016), *Mesembrinella spicata* in Costa Rica and Colombia (Bonatto and Marinoni 2005, Kosmann et al 2013), and *Mesembrinella umbrosa* in Bolivia, Colombia and Ecuador (Guimarães 1977, Peris and Mariluis 1984, Wolff and Kosmann 2016).

The absence from the examined material of species previously recorded in Venezuela or in neighbouring countries reflects the lack of study of these flies in this region. As an example, *Chloroprocta idioidea*, the only species of the genus *Chloroprocta* Wulp, 1896 (Calliphoridae), was recorded in Venezuela by Hall (1948), Cova (1964), Dear (1985) and Kosmann et al. (2013) and was the most abundant (66.3% of the total sampled specimens) species collected in a recent survey of necrophagous flies in the North Brazilian Amazon (Amat et al. 2016). However, it was not found in any Venezuelan museum and only two specimens from Venezuela were examined, from BMNH. In his recent revision, Whitworth (2014) reported *Lucilia albofusca* and *Lucilia vulgata* for Venezuela, but we did not find these two species in our field sampling or in entomological collections. Some authors reported *Lucilia cluvia* and *Lucilia sericata* in Venezuela (Mariluis et al. 1994b, Kosmann et al. 2013), but neither was found during this study. It is unlikely that *Lucilia cluvia* occurs in the country, since Whitworth (2014) argued that reports of this species in South America are incorrect. On the other hand, *Lucilia sericata* has been reported as abundant in neighbouring countries such as Colombia and Brazil (Carvalho and Ribeiro 2000, Pape et al. 2004, Amat et al. 2008), so its absence during this study was surprising. This was also the case of other species previously cited for Venezuela, such as *Compsomyiops fulvicrura*, *Hemilucilia benoisti*, *Paralucilia paraensis*, *Eumesembrinella quadrilineata*, *Eumesembrinella randa*, *Giovanella bolivar*, *Huascaromusca vogelsangi*, *Mesembrinella bellardiana* and *Mesembrinella xanthorrhina* (Aldrich 1922, Shannon 1926, Hall 1948, Cova 1964, Guimarães 1977, Peris and Mariluis 1984, Dear 1985, Peris and Mariluis 1989, Mariluis et al. 1994a, Bonatto and Marinoni 2005, Kosmann et al. 2013). The absence of these species in our samples could be related to non-exhaustive field sampling and/or to the heterogeneous composition of the examined entomological collections.

During this study, some specimens of *Lucilia* and Mesembrinellidae could not be identified as any known species. These specimens may represent undescribed species and for this reason were not included in the checklist. Some species of these groups are morphologically highly variable and further studies are required to determine whether they are separate species or examples of intraspecific variation (Whitworth, pers. comm.). If possible, we strongly recommend rearing specimens from live females collected in the field in order to obtain enough specimens to study the intraspecific variability of both these groups of blow flies.

The distribution maps obtained from the data available (Figs 1–14) show that the current distribution of Venezuelan blow flies is clearly determined by an unequal sampling effort around the country. Most records are from the predominantly mountainous north, where protected areas such as natural parks were the main collection sites. This sampling effort bias makes it difficult to draw any conclusions on the habitat preferences of these species, hence the necessity of further studies. In any case, the presence of some species in areas with less human disturbance adds value to these flies as indicators of the state of habitat conservation. This, in addition to the interest in these species in medicolegal and veterinary fields, will hopefully provide incentive to perform further studies on Venezuelan blow flies.
